# Minimal residual disease assessment by multiparameter flow cytometry in transplant-eligible myeloma in the EMN02/HOVON 95 MM trial

**DOI:** 10.1038/s41408-021-00498-0

**Published:** 2021-06-03

**Authors:** Stefania Oliva, Davine Hofste op Bruinink, Lucie Rihova, Mattia D’Agostino, Lucia Pantani, Andrea Capra, Bronno van der Holt, Rossella Troia, Maria Teresa Petrucci, Tania Villanova, Pavla Vsianska, Romana Jugooa, Claudia Brandt-Hagens, Milena Gilestro, Massimo Offidani, Rossella Ribolla, Monica Galli, Roman Hajek, Francesca Gay, Michele Cavo, Paola Omedé, Vincent H. J. van der Velden, Mario Boccadoro, Pieter Sonneveld

**Affiliations:** 1 Myeloma Unit, Division of Hematology, University of Torino, Azienda Ospedaliero-Universitaria Città della Salute e della Scienza di Torino, Torino, Italy; 2 Department of Hematology, Erasmus MC Cancer Institute, Rotterdam, The Netherlands; 3 Laboratory Medical Immunology, Department of Immunology, Erasmus University Medical Center, Rotterdam, The Netherlands; 4 Department of Clinical Hematology, University Hospital Brno, Brno, Czech Republic; 5 Azienda Ospedaliero-Universitaria di Bologna, Seràgnoli Institute of Hematology, Department of Experimental, Diagnostic and Specialty Medicine, Bologna University School of Medicine, S Orsola Malpighi Hospital, Bologna, Italy; 6 HOVON Data Center, Department of Hematology, Erasmus MC Cancer Institute, Rotterdam, The Netherlands; 7 Hematology, Department of Translational and Precision Medicine, Azienda Ospedaliera Policlinico Umberto I, Sapienza University of Rome, Rome, Italy; 8 Department of Immunology, Laboratory Medical Immunology, Erasmus MC, University Medical Center Rotterdam, Rotterdam, The Netherlands; 9 Clinica di Ematologia, Azienda Ospedaliero Universitaria Ospedali Riuniti Umberto I-G.M. Lancisi-G. Salesi di Ancona, Ancona, Italy; 10 Division of Hematology, ASST Spedali Civili di Brescia, Brescia, Italy; 11 Dipartimento di Oncologia ed Ematologia, ASST Papa Giovanni XXIII, Bergamo, Italy; 12 Department of Haematooncology, University Hospital Ostrava, Ostrava, Czech Republic; 13 Faculty of Medicine, University of Ostrava, Ostrava, Czech Republic

**Keywords:** Risk factors, Translational research

## Abstract

Minimal residual disease (MRD) by multiparameter flow cytometry (MFC) is the most effective tool to define a deep response in multiple myeloma (MM). We conducted an MRD correlative study of the EMN02/HO95 MM phase III trial in newly diagnosed MM patients achieving a suspected complete response before maintenance and every 6 months during maintenance. Patients received high-dose melphalan (HDM) versus bortezomib-melphalan-prednisone (VMP) intensification, followed by bortezomib-lenalidomide-dexamethasone (VRd) versus no consolidation, and lenalidomide maintenance. Bone marrow (BM) samples were processed in three European laboratories, applying EuroFlow-based MFC protocols (eight colors, two tubes) with 10^−4^−10^−5^ sensitivity. At enrollment in the MRD correlative study, 76% (244/321) of patients were MRD-negative. In the intention-to-treat analysis, after a median follow-up of 75 months, 5-year progression-free survival was 66% in MRD-negative versus 31% in MRD-positive patients (HR 0.39; *p* < 0.001), 5-year overall survival was 86% versus 69%, respectively (HR 0.41; *p* < 0.001). MRD negativity was associated with reduced risk of progression or death in all subgroups, including ISS-III (HR 0.37) and high-risk fluorescence in situ hybridization (FISH) patients (HR 0.38;). In the 1-year maintenance MRD population, 42% of MRD-positive patients at pre-maintenance became MRD-negative after lenalidomide exposure. In conclusion, MRD by MFC is a strong prognostic factor. Lenalidomide maintenance further improved MRD-negativity rate.

## Introduction

Multiple myeloma (MM) treatment has considerably improved in the past 15–20 years. The current paradigm for transplant-eligible newly diagnosed MM (NDMM) patients consists of induction, stem-cell mobilization and autologous stem-cell transplantation (ASCT), followed by consolidation and/or maintenance. With this approach, more than 60% of patients can achieve a complete response (CR), which has historically been considered one of the most powerful prognostic factors in MM^1^.

Currently, minimal residual disease (MRD) assessment is the most sensitive tool to measure the depth of response in MM patients. Indeed, among patients achieving a CR, MRD-positive patients have an inferior progression-free survival (PFS) and overall survival (OS) compared with MRD-negative ones^2^, and a similar outcome to those achieving a partial response (PR)^3^. Therefore, MRD assessment has been introduced in the International Myeloma Working Group (IMWG) response criteria since 2011, making CR no longer the most reliable clinical endpoint.

While PFS may require many years to demonstrate the effectiveness of a new treatment strategy^4,5^, MRD evaluation can be a quicker tool to show the clinical benefit of a treatment and obtain its approval. Therefore, several studies have suggested that MRD negativity can be used as a surrogate endpoint for both PFS and OS.

To investigate this in the context of a large clinical trial, we evaluated MRD by multiparameter flow cytometry (MFC) as a predictor of PFS and OS in a large cohort of NDMM patients enrolled in the EMN02/HO95 MM phase III trial. We also assessed the role of continuous MRD monitoring during lenalidomide maintenance, and the prognostic value of maintaining MRD negativity.

## Methods

### Patients and clinical trial

**Fig. 1:**
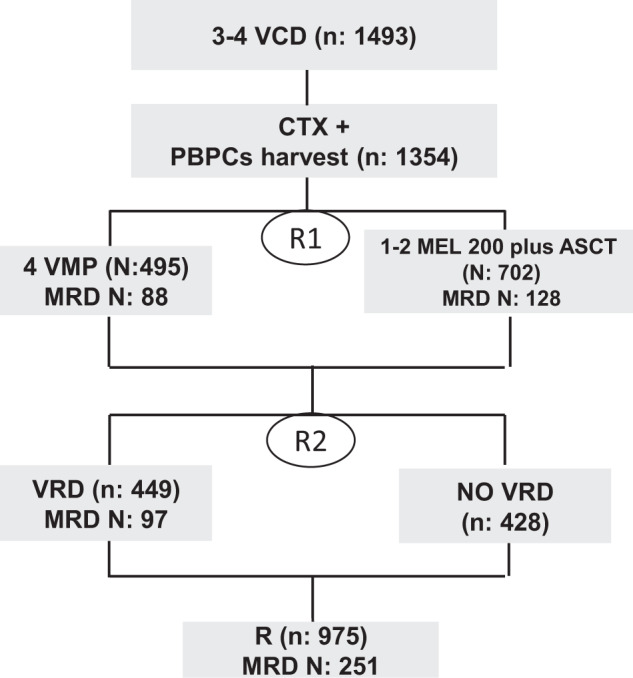
Study design. VCd, bortezomib-cyclophosphamide-dexamethasone; VMP, bortezomib-melphalan-prednisone; ASCT, autologous stem-cell transplantation; VRD, bortezomib-lenalidomide-dexamethasone; CTX, cyclophosphamide; PBPCs, peripheral blood plasma cells; R1, first randomization; R2, second randomization; R, lenalidomide; MRD, minimal residual disease.

Clinical results of the EMN02/HO95 MM phase III trial have been published previously^6^. Briefly, transplant-eligible patients aged ≤65 years were enrolled from February 2011 to April 2014 in 172 European centers (European Myeloma Network [EMN]) and received three to four cycles of bortezomib-cyclophosphamide-dexamethasone (VCd) induction followed by mobilization and stem-cell collection. Patients were first randomized to intensification treatment with four cycles of bortezomib-melphalan-prednisone (VMP) versus high-dose melphalan (HDM) followed by autologous stem-cell transplantation (ASCT); a second randomization was performed between consolidation with bortezomib-lenalidomide-dexamethasone (VRd) versus no consolidation. Finally, patients received lenalidomide maintenance until progression or intolerance (Fig. 1). Randomization (1:1 ratio) was stratified according to site and International Staging System (ISS) disease stage. In centers with a double transplantation policy, patients were randomized (1:1:1) to VMP or single transplantation or double transplantation.

All patients provided written informed consent before enrollment. The study was approved by the independent ethics committees or institutional review boards at each participating site and was conducted in accordance with the Declaration of Helsinki and registered at ClinicalTrials.gov (NCT01208766).

### Minimal residual disease detection by multiparameter flow cytometry

MFC MRD status was assessed in patients with a suspected CR (100% reduction of serum and/or urine M-component) before starting maintenance (after HDM, VMP, or VRD) and every 6 months during maintenance until progressive disease (PD). Patients provided specific written informed consent for MRD analyses.

MFC was performed on BM aspirates according to EuroFlow-based methods (eight colors, two tubes) for sample processing and cell acquisition^7,8^. Samples were obtained in ten countries and subsequently processed and analyzed in three centralized European laboratories (Torino, Italy; Brno, Czech Republic; Rotterdam, the Netherlands). Two laboratories (Brno and Rotterdam) applied the EuroFlow PCD panel; data were acquired using a FACSCanto II (BD) flow cytometer and analyzed with Infinicyt 1.7 software (Cytognos). One of the laboratories (Torino) applied a local panel with minor differences in fluorochromes and antibodies; data were acquired using a Navios flow cytometer and analyzed with Kaluza software (Beckman Coulter, Brea, US-CA). Specific MFC panels are shown in the Supplementary Material and have been published previously^9^.

We aimed to acquire at least 2 million cells. The cutoff for MRD positivity was set at ≥20 clonal plasma cells out of the total of nucleated cells, resulting in a general sensitivity between 10^−4^ and 10^−5^.

### Statistical analysis

The association between MRD status before and during maintenance therapy and survival endpoints was assessed in four different patient populations:

i. A *modified intention-to-treat (ITT) population*, including all patients that were eligible for the main analysis of the study, and excluded those achieving a CR or stringent CR (sCR) but with no sample available for MRD analysis at any timepoint^10^. Patients with ≤very good partial response (VGPR) were considered MRD-positive.
ii. A *pre-maintenance MRD population*, including patients with an available MRD sample before starting maintenance or within the first 4 months after the start of maintenance. For patients with ≥2 available samples, the last evaluation was chosen. Patients with an available sample only after induction or mobilization were excluded from this analysis.
iii. A *sustained MRD-negative population*, including patients with MRD negativity confirmed in two samples obtained at least 1 year apart, at any time in the treatment protocol.
iv. A *1-year maintenance MRD population*, including patients with availability of an MRD sample at 12 months after the start of maintenance (±3 months).

PFS and OS were analyzed using the Kaplan–Meier method in the pre-maintenance MRD population and the Kaplan–Meier method modified by the Simon–Makuch method in the other populations^11^. PFS was defined as the time from MRD correlative study entry or informed consent date to PD or death from any cause. OS was defined as the time from MRD correlative study entry or informed consent date to death from any cause. The multivariate Cox model, including MRD as a fixed covariate or as a time-dependent covariate, was used to estimate hazard ratios (HRs) and 95% confidence intervals (CIs). Patients who went off protocol due to withdrawal of consent were excluded from the analysis. The concordance between MRD results of the different laboratories was assessed through proportions of disagreements and Cohen’s kappa coefficient (*κ*). Data were analyzed using R Software (v3.5.1). Data cutoff was 5 February 2020.

## Results

### Patient characteristics and therapies

First, we checked the technique sensitivity and verified the concordance of the MFC protocols used in the three European laboratories. Ten samples from MM patients enrolled in EMN02/HO95 MM phase III trial were simultaneously analyzed by the three laboratories and data results were highly concordant^9^. To further support this, 100 MRD FACS files from the three laboratories were retrospectively analyzed and the concordance was high (90%, *κ* = 0.81, 95% CI 0.74–0.87; Supplementary Table 1)

A total of 321 patients before lenalidomide maintenance were evaluated for MRD assessment. Patient characteristics at registration in the trial are listed in Table 1: median age was 57 years (IQR: 52–62); 55 (17%) had ISS-III; 69 (26%) had high-risk cytogenetic abnormalities by FISH, defined as the presence of at least one of del17p, *t*(4;14) and/or *t*(14;16); 45 (15%) had lactate dehydrogenase (LDH) > upper limit of normal; 24 (7%) had Revised ISS (R-ISS) stage III. Patient characteristics were consistent with the main EMN02/HOVON 95 MM study^6^.

**Table 1:** Patient characteristics of pre-maintenance evaluable population.

	*N* = 321
Age (years)	
Median (IQR)	57 (52–62)
Gender *N* (%)	
Male	180 (56)
ISS *N* (%)	
I	143 (45)
II	123 (38)
III	55 (17)
R-ISS *N* (%)	
I	82 (26)
II	177 (55)
III	24 (7)
Missing	38 (12)
LDH *N* (%)	
>ULN	45 (15)
Missing data *N* (%)	16 (5)
Cytogenetic features *N* (%)	
Deletion 17p	25 (9)
Translocation (4;14)	40 (15)
Translocation (14;16)	11 (4)
High risk	69 (26)
Missing data	55 (17)
Random *N* (%)	
No ASCT	119 (37)
ASCT	202 (63)

*N* number, *IQR* interquartile range, *ISS* International Staging System, *R-ISS* Revised ISS, *LDH* lactate dehydrogenase, *ULN* upper limit of normal, *ASCT* autologous stem-cell transplantation.

A total of 202 (63%) patients received HDM and 119 (37%) VMP; 158 (49%) patients received VRD consolidation according to protocol. MRD data were available for 248 patients in CR/sCR (77%) and 73 with unconfirmed-CR (lack of data about serum or urine immunofixation)/VGPR (23%). At MRD enrollment, 76% of patients (244/321) were MRD-negative: 64% (157/244) in the HDM versus 36% (87/244) in the VMP groups. A total of 1204 samples were analyzed at different time-points for MRD, with a median limit of detection (LOD) of 0.001% (IQR 0.0006%–0.002%).

### Modified ITT analysis

**Fig. 2:**
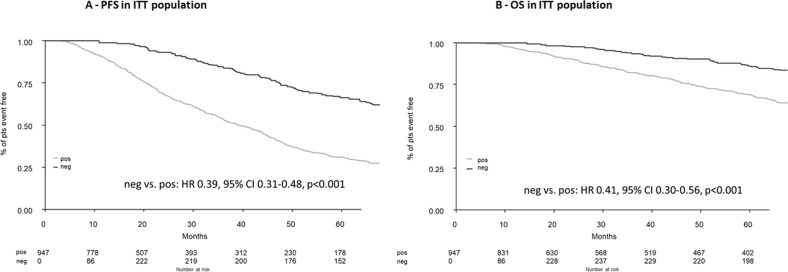
PFS, progression-free survival; OS, overall survival; ITT, intention to treat; pos, positive; neg, negative; pts, patients. **A** Progression-free survival in the intention-to-treat population. **B** Overall survival in the intention-to-treat population.

**Fig. 3:**
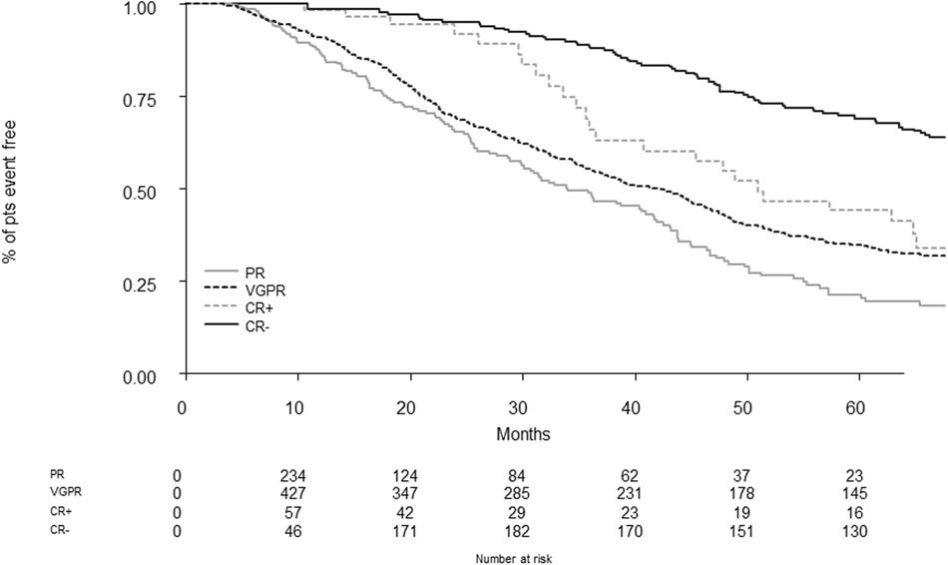
PR, partial response; VGPR, very good partial response; CR, complete response; pts, patients. Progression-free survival in the intention-to-treat population (best overall) stratified by response.

**Fig. 4:**
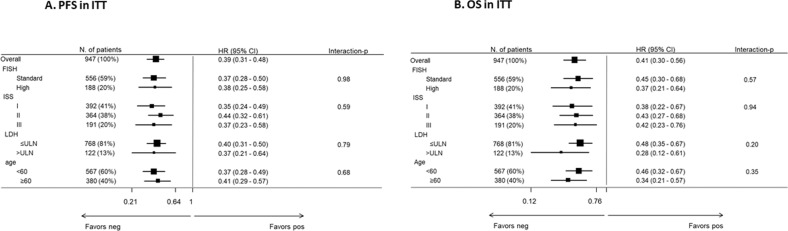
FISH, fluorescence in situ hybridization; ISS, International Staging System; LDH, lactate dehydrogenase; ULN, upper limit of normal; PFS, progression-free survival; OS, overall survival; ITT, intention-to-treat. **A** Subgroup analysis of progression-free survival in the intention-to-treat population. **B** Subgroup analysis of overall survival in the intention-to-treat population.

The modified ITT analysis included a total of 947/1197 (79%) patients who underwent the first randomization in the EMN02/HOVON95 MM clinical trial; 250 ≥CR (as best response) patients were excluded due to missing MRD data. To exclude potential bias, we compared baseline clinical features and outcomes between patients in CR with versus without MRD data and we did not observe any difference (Supplementary Table 2). After a median follow-up of 75 months (IQR 66–83 months), 5-year PFS was 66% in MRD-negative versus 31% in MRD-positive patients (median: 92 versus 39 months; HR 0.39, 95% CI 0.31–0.48, *p* < 0.001); the respective 5-year OS was 86% versus 69% (HR 0.41, 95% CI 0.30–0.56, *p* < 0.001) (Fig. 2) Importantly, the achievement of MRD negativity dissected a good prognostic CR population when compared to other IMWG responses (Fig. 3). The multivariable Cox analysis showed that MRD, ISS, FISH and LDH had an independent prognostic value for PFS and OS, and confirmed MRD as the most significant prognostic marker (Table 2). Subgroup analyses were performed to determine the consistency of effects of MRD negativity versus positivity in the different subgroups, using interaction-p terms between each of the covariates included in the Cox model. MRD negativity reduced the risk of progression or death in all subgroups (Fig. 4).

**Table 2:** Multivariate Cox analysis ITT.

Progression-free survival				Overall survival			
	HR	95% CI	*p*-value		HR	95% CI	*p*- value
MRD status: neg versus pos	0.39	(0.31–0.48)	<0.001	MRD status: neg versus pos	0.41	(0.30–0.56)	<0.001
FISH: High versus Standard	1.57	(1.29–1.92)	<0.001	FISH: High versus Standard	2.29	(1.79–2.95)	<0.001
ISS: II versus I	1.29	(1.08–1.54)	0.006	ISS: II versus I	1.61	(1.24–2.08)	<0.001
ISS: III versus I	1.72	(1.39–2.12)	<0.001	ISS: III versus I	2.32	(1.74–3.08)	<0.001
LDH: > ULN versus ≤ULN	1.39	(1.10–1.75)	0.005	LDH: > ULN versus ≤ULN	1.82	(1.37–2.42)	<0.001
Age: ≥60 versus <60	0.97	(0.83–1.14)	0.741	Age: ≥60 versus <60	1.04	(0.84–1.30)	0.696

Adjusted for sex.

*MRD* minimal residual disease, *FISH* fluorescence in situ hybridization, *ISS* International Staging System, *LDH* lactate dehydrogenase, *ULN* upper limit of normal, *ITT* intention to treat.

**Fig. 5:**
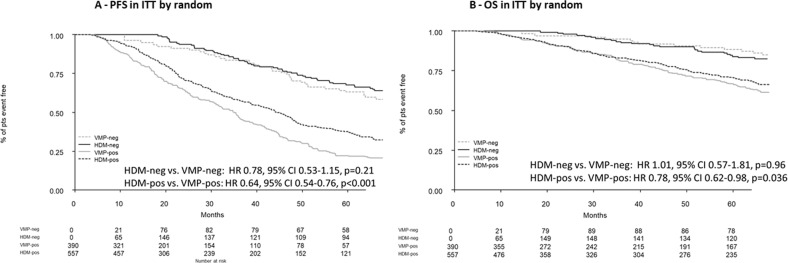
HDM, high-dose melphalan; VMP, bortezomib-melphalan-prednisone; PFS, progression-free survival; OS, overall survival; ITT, intention-to-treat; pts, patients. **A** Progression-free survival in the intention-to-treat population by treatment. **B** Overall survival in the intention-to-treat population by treatment.

The prognostic impact of MRD negativity on PFS was independent of previous intensification treatment: median PFS was NR for HDM versus 88 months for VMP (HR 0.78, 95% CI 0.53–1.15, *p* = 0.21); whereas, in MRD-positive patients, median PFS was 44 months with HDM versus 35 months with VMP (HR 0.64, 95% CI 0.54–0.76, *p* < 0.001). Median OS was NR for HDM and VMP (HR 1.01, 95% CI 0.57–1.81, *p* = 0.96); whereas, in MRD-positive patients, median OS was 95 months with HDM versus 78 months with VMP (HR 0.78, 95% CI 0.62–0.98, *p* = 0.036) (Fig. 5). Moreover, the prognostic impact of MRD negativity was independent from the three different laboratories (Turin versus Brno/Rotterdam) both on PFS (HR: 1.12, 95% CI 0.75–1.67, *p* = 0.57) and OS (HR: 1.05, 95% CI: 0.59–1.87, *p* = 0.86) to confirm no major technical bias.

### Pre-maintenance population outcome

After a median follow-up of 64 months from the time of pre-maintenance sampling, the 5-year PFS was 57% in MRD-negative versus 32% in MRD-positive patients (median: 87 versus 38 months; HR 0.39, 95% CI: 0.28–0.56, *p* < 0.001). The 5-year OS was 82% in MRD-negative versus 69% in MRD-positive patients (median: NR in both groups; HR 0.51, 95% CI 0.31–0.85, *p* = 0.01) (Supplementary Fig. 1). The multivariable Cox analysis showed that MRD negativity, ISS and high-risk FISH (HR-FISH) had an independent association with PFS and OS, and confirmed MRD negativity and HR-FISH were the most significant prognostic markers (Supplementary Table 3). MRD negativity reduced the risk of progression or death in ISS-I (HR 0.48), ISS-II (HR 0.47) and ISS-III (HR 0.11, interaction-*p* = 0.008), as well as in standard-risk (HR 0.60) and HR-FISH patients (HR 0.15, interaction-*p* < 0.001; Supplementary Fig. 2) supporting MRD negativity as one of the most relevant clinical endpoints in MM patients, particularly in the high-risk setting.

In patients with high-risk disease by FISH and with ISS-III, MRD positivity at pre-maintenance was associated with a dismal outcome compared to MRD negativity. In the HR-FISH group, median PFS was 15 months for MRD-positive versus 53 months for MRD-negative patients (HR 0.18, 95% CI 0.09–0.35, *p* < 0.001), and the respective median OS was 44 months versus not reached (HR 0.23 95% CI 0.11–0.51; *p* < 0.001). In the ISS-III group, median PFS was 7 months for MRD-positive versus 67 months for MRD-negative subjects (HR 0.12, 95% CI 0.05–0.31; *p* < 0.001) and the respective median OS was 18 months versus not reached (HR 0.14, 95% CI 0.05–0.37; *p* < 0.001) (Supplementary Fig. 3).

Patients with sustained MRD negativity had a 5-year PFS of 81% and 5-year OS of 94%, (Supplementary Fig. 4).

### One-year lenalidomide maintenance MRD analysis and MRD conversion

An MRD analysis at 1 year of lenalidomide maintenance was performed in 118 patients: 5-year PFS was 79% in 87 MRD-negative versus 48% in 31 MRD-positive patients (median: 98 versus 57 months; HR 0.32, 95% CI 0.16–0.62, *p* < 0.001); 5-year OS was 95% in MRD-negative versus 82 % in MRD-positive patients (median: NR in both groups; HR 0.37, 95% CI 0.12–1.14, *p* = 0.08) (Supplementary Fig. 5).

Moreover, the impact of MRD status conversion was evaluated in this 1-year lenalidomide maintenance population (Supplementary Fig. 6): ten patients who converted from MRD-positive to MRD-negative did not have a significant different PFS to those with persistent negativity (*N* = 77) during lenalidomide treatment (5-year PFS 90% versus 77%, respectively, HR 0.78, 95% CI 0.16–3.73, *p* = 0.75). Conversely, 17 patients who were MRD-negative and became MRD-positive showed a higher risk of progression, similar to those with persistent positivity (*N* = 14) (5-year PFS 54% versus 34%, respectively, HR 0.75, 95% CI 0.26–2.17, *p* = 0.595). However, 3/14 (21%) persistent MRD-positive patients did not experience disease relapse at data cutoff.

We evaluated MRD conversion rate during maintenance in this subset of patients: 10/24 (41%) patients converted from MRD-positive to MRD-negative when receiving lenalidomide. Of these patients, 8/24 (33%) changed their MRD status within 6–12 months and 2/24 (8%) after 18 months from start of lenalidomide maintenance, with 7/10 (70%) receiving VRD consolidation. Importantly, all 10 patients who experienced MRD conversion presented with standard-risk FISH at baseline, LDH < ULN and ISS-I or -II.

## Discussion

Several clinical trials and two meta-analyses evidenced the role of MRD as a possible surrogate for survival in NDMM. Indeed, MRD negativity was associated with a benefit in terms of PFS and OS compared with MRD positivity, dissecting CR patients into two distinct populations with different outcomes^2,12^.

In our study, we confirmed the prognostic role of MRD negativity in a large cohort of NDMM patients. In the pre-maintenance population, after a median follow-up of 64 months, we observed a significant PFS difference between MRD-negative and MRD-positive patients with a suspected CR (HR 0.39, *p* < 0.001), and we confirmed that MRD negativity, whenever achieved, is associated with a significantly improved outcome. These results were obtained in a really international context—thus confirming their solid nature—and are in line with those reported by other groups (IFM, MRC)^10^. Nevertheless, MRD was an optional sub-study of the EMN02 trial, and the high number of missing data affected a precise evaluation of different MRD-negative rates in the different arms and a correlation of MRD rates with PFS. MRD assessment was performed in three different European laboratories: despite some methodology differences, the initial simultaneous evaluation of ten random samples and the subsequent blinded analysis of 100 MRD FACS files (90% of overall concordance) by the three laboratories confirmed no major technical bias. This is particularly important for future MRD studies involving various countries. Indeed, by following international guidelines, results from different laboratories can still be highly concordant.

By longitudinal MRD monitoring, patients improving from MRD-positive to MRD-negative status had a similar outcome to those with persistent MRD negativity. Conversely, patients who converted from MRD-negative to MRD-positive had a higher risk of relapse, like patients with persistent MRD positivity. Nonetheless, a small proportion of persistent MRD-positive patients did not progress at the data cutoff, suggesting that an MGUS-like indolent phenotype could still be present and thus changing treatment should be contraindicated. Moreover, a precise immune profiling by flow cytometry can offer complementary information to the simple quantification of MRD levels and may contribute to identifying a subset of patients that, albeit being MRD-positive, can still experience prolonged survival due to a unique immune signature (ex: with a more prominent regeneration of mature B lymphocytes) with probably a competent immune surveillance keeping myeloma burden in repression^13,14^. It would be informative to evaluate the genomic profile of these residual MM cells or to monitor the trend of MRD kinetics, as previously done for other hematologic diseases^15–19^. This could help to identify patients who would benefit from an early change in treatment strategy.

Lenalidomide maintenance after ASCT is nowadays considered standard of care for NDMM patients^20–24^. Still, the optimal duration of treatment is a matter of debate, raising the question whether continuous maintenance therapy with lenalidomide is equally beneficial for all patients or if maintenance treatment should be of limited duration in some patients, to allow true treatment-free intervals. The GMMG-MM5 phase III trial showed that lenalidomide maintenance after ASCT or consolidation should be applied beyond achievement of a CR for 2 years, since therapy interruption in CR patients was associated with a shorter PFS in comparison with continuous treatment (HR 1.84, *p* = 0.02)^25^. Preliminary MRD data from the MRC XI trial showed that lenalidomide maintenance for 2 years prolonged PFS, independently of MRD status^26^. In our study, lenalidomide maintenance improved MRD negativity rate by 41%, and patients with sustained MRD negativity during maintenance treatment continued to have a good PFS and OS. This suggests that lenalidomide maintenance treatment is able to convert MRD status and further deepens responses during the first 6–12 months of therapy. Consequently, if MRD negativity is a primary clinical endpoint, and MRD-positive status does not convert to MRD-negative within the first year of lenalidomide maintenance, a treatment change should be suggested. Of note, this can be a valid strategy to balance efficacy versus toxicity and may favor fixed-duration therapy in this setting. Alonso and colleagues showed that serial MRD monitoring during maintenance could be important to evaluate the deepening of response, with 34% of patients who converted from positive to negative after a median time of 18 months^27^. Still, in our study, we observed a subgroup of patients who converted to negativity after 18 months (8%). The rate of MRD conversion from positive to negative with lenalidomide maintenance was remarkable in our study, similarly to the MRC XI (32%) and BMT CTN 0702 (30%) trials^26,28^. Yet, this rate was much lower in the PETHEMA/GEM2012MENOS65 trial (17%). This could be related to the previous lenalidomide-based induction treatment used in the Spanish study, which might have affected patients’ sensitivity to lenalidomide maintenance^29–31^. Therefore, different in-class drugs than those used during the induction phase should be suggested in the maintenance setting. Future prospective randomized trials are needed to further compare continuous lenalidomide maintenance with fixed-duration therapy (1 or 2 years) in patients who are MRD-negative at a fixed timepoint.

Despite the prognostic significance of MRD, some patients still relapse. In our study this might be due firstly to the MFC method used, which is a little less sensitive as compared with more recent techniques, such as next-generation flow (NGF) protocols (EuroFlow) and next-generation sequencing (NGS). Recently, Paiva et al.^29^ have confirmed that 10^−6^ by NGF should be considered a clinically significant cutoff for the achievement of MRD negativity by flow cytometry, with only 7% of patients who relapsed, mostly with extramedullary disease. Our study has a lower sensitivity (10^-4^−10^-5^) since it was initiated in 2011, when the newer, more sophisticated techniques were not available. Secondly, in our study no paired bone marrow-positron emission tomography/computed tomography (BM-PET/CT) data were available to detect extramedullary relapses. Several studies showed that MRD negativity confirmed by both flow cytometry and functional imaging (double negative) can identify a better prognosis population compared with MRD negativity detected only in the BM marrow^32,33^. In the CASSIOPEIA trial, patients treated with daratumumab-based strategies showed a low concordance between PET/CT and MRD in the BM, supporting the concept that imaging should always be matched with BM techniques, at least for patients with extramedullary disease. Moreover, patients who were double negative after consolidation showed a better PFS^34^.

MRD evaluation is a fundamental endpoint in MM, particularly for high-risk patients by FISH, ISS, and R-ISS. In patients with high-risk disease at diagnosis and persistent residual disease, an intensified treatment adding a new drug or changing treatment earlier should be preferred. In this “difficult-to-treat” population, novel therapies, such as CAR-T or bispecific antibodies, could be potentially effective strategies, as shown in recent studies in the RRMM stetting^35,36^. Newer quadruplet regimens are being explored in NDMM patients receiving ASCT. The CASSIOPEIA and GRIFFIN studies showed that the addition of antiCD38 to standard regimens (VTD or VRD) improves MRD negativity; interestingly, quadruplets improve sCR rates across most of the MM subgroups, except for patients with ISS stage III disease or high-risk cytogenetics^37,38^. However, in the Griffin study, the subgroup analysis for MRD negativity showed that daratumumab-VRD favored all subgroups, including high-risk patients. In the MASTER trial, daratumumab plus carfilzomib-lenalidomide-dexamethasone (Dara-KRd) after consolidation induced a high rate of MRD negativity at 10^−5^ (83%), even in patients with high-risk FISH^39^. In addition, we found that lenalidomide treatment converted MRD positivity to negativity particularly in the standard-risk group, further confirming the need to switch to intensified strategies in high-risk MRD-positive patients. Conversely, we found that patients with high-risk disease in suspected CR and without persistent MRD after treatment have a very favorable outcome, since MRD negativity can overcome the poor impact of MM high-risk features in these CR patients. Although our study used standard treatment strategies and no newer quadruplets, achieving MRD negativity was still crucial and independent from previous intensification treatment (VMP or HDM), and the long follow-up increased the value of our analyses.

In conclusion, our study confirms that MRD status by MFC is a strong prognostic factor in NDMM patients receiving intensification with novel agents or HDM. The achievement of MRD negativity in patients with HR-FISH aberrations was associated with a significantly improved survival, underlining the importance of achieving deep responses in this setting. Finally, lenalidomide maintenance further improved the depth of response in standard-risk patients.

## Supplementary information

Supplementary Material

## References

[1] D’Agostino M., Bertamini L., Oliva S., Boccadoro M. & Gay F. Pursuing a curative approach in multiple myeloma: a review of new therapeutic strategies. *Cancers (Basel)* **11**, 10.3390/cancers11122015 (2019). 10.3390/cancers11122015 PMC6966449 31847174

[2] Munshi NC (2017). Association of minimal residual disease with superior survival outcomes in patients with multiple myeloma: a meta-analysis. JAMA Oncol..

[3] Lahuerta J-J (2017). Depth of response in multiple myeloma: a pooled analysis of three PETHEMA/GEM clinical trials. J. Clin. Oncol..

[4] Kumar S (2016). International Myeloma Working Group consensus criteria for response and minimal residual disease assessment in multiple myeloma. Lancet Oncol..

[5] Landgren O, Owen RG (2016). Better therapy requires better response evaluation: paving the way for minimal residual disease testing for every myeloma patient. Cytom. Part B Clin. Cytom..

[6] Cavo M (2020). Autologous haematopoietic stem-cell transplantation versus bortezomib–melphalan–prednisone, with or without bortezomib–lenalidomide–dexamethasone consolidation therapy, and lenalidomide maintenance for newly diagnosed multiple myeloma (EMN02/HO95): a mult. Lancet Haematol.

[7] Kalina T (2012). EuroFlow standardization of flow cytometer instrument settings and immunophenotyping protocols. Leukemia.

[8] van Dongen JJM (2012). EuroFlow antibody panels for standardized n-dimensional flow cytometric immunophenotyping of normal, reactive and malignant leukocytes. Leukemia.

[9] Hofste op Bruinink, D. et al. Standardization of flow cytometric minimal residual disease assessment in 2 international clinical trials–a feasibility study from the European Myeloma Network. *Haematologica*10.3324/haematol.2020.267831 (2021). 10.3324/haematol.2020.267831 PMC8094105 33054135

[10] Perrot A (2018). Minimal residual disease negativity using deep sequencing is a major prognostic factor in multiple myeloma. Blood.

[11] Simon R, Makuch RW (1984). A non‐parametric graphical representation of the relationship between survival and the occurrence of an event: application to responder versus non‐responder bias. Stat. Med..

[12] Landgren O, Devlin S, Boulad M, Mailankody S (2016). Role of MRD status in relation to clinical outcomes in newly diagnosed multiple myeloma patients: a meta-analysis. Bone Marrow Transplant.

[13] Paiva B (2016). Minimal residual disease monitoring and immune profiling in multiple myeloma in elderly patients. Blood.

[14] Papadimitriou K (2020). Deep phenotyping reveals distinct immune signatures correlating with prognostication, treatment responses, and MRD status in multiple myeloma. Cancers (Basel).

[15] Paiva B (2013). A multiparameter flow cytometry immunophenotypic algorithm for the identification of newly diagnosed symptomatic myeloma with an MGUS-like signature and long-term disease control. Leukemia.

[16] Michor F (2005). Dynamics of chronic myeloid leukaemia. Nature.

[17] Tang M (2011). Dynamics of chronic myeloid leukemia response to long-term targeted therapy reveal treatment effects on leukemic stem cells. Blood.

[18] Hoffmann H (2019). The prognostic potential of monitoring disease dynamics in NPM1-positive acute myeloid leukemia. Leukemia.

[19] Lee S (2012). Impact of minimal residual disease kinetics during imatinib-based treatment on transplantation outcome in Philadelphia chromosome-positive acute lymphoblastic leukemia. Leukemia.

[20] Attal M (2017). Lenalidomide, bortezomib, and dexamethasone with transplantation for myeloma. N. Engl J. Med..

[21] Palumbo A (2014). Autologous transplantation and maintenance therapy in multiple myeloma. N. Engl J. Med..

[22] McCarthy PL (2012). Lenalidomide after stem-cell transplantation for multiple myeloma. N. Engl J. Med..

[23] Attal M (2012). Lenalidomide maintenance after stem-cell transplantation for multiple myeloma. N. Engl J. Med..

[24] Palumbo A (2012). Continuous lenalidomide treatment for newly diagnosed multiple myeloma. N. Engl J. Med..

[25] Goldschmidt H (2020). Response-adapted lenalidomide maintenance in newly diagnosed myeloma: results from the phase III GMMG-MM5 trial. Leukemia.

[26] de Tute, R. M. et al. Minimal residual disease in the maintenance setting in myeloma: prognostic significance and impact of lenalidomide. *Blood* **130**, Abstract #904 [ASH 2017 59th Meeting] (2017).

[27] Alonso R (2020). Prolonged lenalidomide maintenance therapy improves the depth of response in multiple myeloma. Blood Adv..

[28] Hahn TE (2019). Minimal residual disease (MRD) assessment before and after autologous hematopoietic cell transplantation (AutoHCT) and maintenance for multiple myeloma (MM): results of the prognostic immunophenotyping for myeloma response (PRIMeR) Study. Biol. Blood Marrow Transplant.

[29] Paiva B (2020). Measurable residual disease by next-generation flow cytometry in multiple myeloma. J. Clin. Oncol..

[30] De Tute R (2019). Sequential minimal residual disease (MRD) monitoring: results from the UK Myeloma XI trial. Clin. Lymphoma Myeloma Leuk..

[31] Stadtmauer EA (2019). Autologous transplantation, consolidation, and maintenance therapy in multiple myeloma: Results of the BMT CTN 0702 trial. J. Clin. Oncol..

[32] Moreau P (2017). Prospective evaluation of magnetic resonance imaging and [18F]fluorodeoxyglucose positron emission tomography-computed tomography at diagnosis and before maintenance therapy in symptomatic patients with multiple myeloma included in the IFM/DFCI 2009 Trial. J. Clin. Oncol..

[33] Rasche L (2019). Combination of flow cytometry and functional imaging for monitoring of residual disease in myeloma. Leukemia.

[34] Moreau P (2019). Evaluation of the prognostic value of positron emission tomography-computed tomography (PET-CT) at diagnosis and follow-up in transplant-eligible newly diagnosed multiple myeloma (te ndmm) patients treated in the phase 3 cassiopeia study: results of the cassiopet companion study. Blood.

[35] Madduri, D. et al. Results from CARTITUDE-1: a phase 1b/2 study of JNJ-4528, a CAR-T cell therapy directed against B-Cell maturation antigen (BCMA), in patients with relapsed and/or refractory multiple myeloma (R/R MM). *Blood* **134**, Abstract #577 [ASH 2019 61st Annual Meeting] (2019).

[36] Munshi NC (2020). Idecabtagene vicleucel (ide-cel; bb2121), a BCMA-targeted CAR T-cell therapy, in patients with relapsed and refractory multiple myeloma (RRMM): Initial KarMMa results. J. Clin. Oncol..

[37] Moreau P (2019). Bortezomib, thalidomide, and dexamethasone with or without daratumumab before and after autologous stem-cell transplantation for newly diagnosed multiple myeloma (CASSIOPEIA): a randomised, open-label, phase 3 study. Lancet.

[38] Voorhees PM (2020). Daratumumab, lenalidomide, bortezomib, and dexamethasone for transplant-eligible newly diagnosed multiple myeloma: the GRIFFIN trial. Blood.

[39] Costa, L. J. et al. First clinical study of the B-cell maturation antigen (BCMA) 2+1 T cell engager (TCE) CC-93269 in patients (Pts) with relapsed/refractory multiple myeloma (RRMM): interim results of a phase 1 multicenter trial. *Blood* **134**, Abstract #143 [updated results presented at ASH 2019] (2019).

