# Supplementary material for: Minimal residual disease assessment by multiparameter flow cytometry in transplant-eligible myeloma in the EMN02/HOVON 95 MM trial
Source: Blood Cancer J. 2021 Jun 3;11(6):106. doi: 10.1038/s41408-021-00498-0 (PMC8175611; doi:10.1038/s41408-021-00498-0)
Supplement: Supplementary file 1 — Supplementary Material [file 41408_2021_498_MOESM1_ESM.pdf]

Supplementary Material

Flow panels in the 3 laboratories:

- **Turin:**

  tube 1: CD81FITC/CD27PE/CD138PC5.5/CD19PE-Cy7/CD20APC/CD38PB/CD45KO;

  tube 2: cyKappaFITC/cyLambdaPE/CD138PC5.5/CD19PE-Cy7/CD56APC/CD117APC-A750/CD38PB/CD45KO)

- **Brno:**

  tube 1: CD81APC-H7/CD27PerCP-Cy5.5/CD138PO/CD19PE-Cy7/ CD28PE/ CD38FITC/CD45PB/CD117APC;

  tube 2: cyKappaAPC/cyLambdaAPC-H7/CD138PO/CD19PE-Cy7/ CD56PE/ CD38FITC/CD45PB/β2micro-APC

- **Rotterdam:**

  tube 1: CD81APC-H7/CD27PerCP-Cy5.5/CD138PO/CD19PE-Cy7/ CD28PE/ CD38FITC/CD45PB/CD117APC;

  tube 2: cyKappaAPC/cyLambdaAPC-C750/CD138PO/CD19PE-Cy7/ CD56PE/ CD38FITC/CD45PB/β2micro-APC)

Supplementary Table 1. Overall correlation among 100 FACS files in the 3 laboratories

	Neg	Pos
**Neg**	136	18
**Pos**	11	133

Supplementary Table 2. Baseline characteristics of MRD evaluable-CR patients vs MRD not evaluable-CR patients

	MRD-CR N=248	No MRD-CR N=250	P value
**Age (years)**			
Median (IQR)	57 (52 - 62)	57 (53 - 62)	0.73
**Gender, N (%)**			
Male	137 (55)	128 (51)	0.37
**ISS, N (%)**			
I	112 (45)	103 (41)	0.31
II	99 (40)	97 (39)	
III	37 (15)	50 (20)	
**R-ISS, N (%)**			
I	67 (30)	47 (23)	0.26
II	141 (63)	139 (68)	
III	15 (7)	17 (8)	
missing	25	47	
**LDH, N (%)**			
> ULN	33 (14)	30 (13)	0.89
Missing data N (%)	13	24	
**Cytogenetic features, N (%)**			
Deletion 17p	19 (9)	21 (11)	0.50
Translocation (4;14)	31 (15)	20 (10)	0.23
Translocation (14;16)	9 (4)	8 (4)	1
High risk	52 (25)	45 (25)	0.90
**Random, N (%)**			
no ASCT	94 (38)	105 (42)	0.36
ASCT	154 (62)	145 (58)	
**PFS*(HR 95% CI)**	MRD-CR vs No MRD CR: 0.91 (0.69-1.20)		0.51
**OS*(HR 95% CI)**	MRD-CR vs No MRD CR: 1.01 (0.65-1.58)		0.95

ISS, International Staging System. R-ISS, Revised International Staging System. LDH, lactate dehydrogenase. ASCT, autologous stem-cell transplantation. MRD, minimal residual disease. CR, complete response. PFS, progression-free survival. OS, overall survival. HR, hazard ratio. CI, confidence interval. *MRD-CR vs no MRD-CR.

Supplementary Table 3. Multivariate Cox analysis

	Progression-free survival					Overall survival			
**Pre-maintenance**		**HR**	**95% CI**	**p value**			**HR**	**95% CI**	**p value**
	MRD status: neg vs pos	0.39	(0.28 - 0.56)	<0.001		MRD status: neg vs pos	0.51	(0.31 - 0.85)	0.01
	FISH: High vs Standard	2.35	(1.61 - 3.44)	<0.001		FISH: High vs Standard	2.46	(1.46 - 4.13)	<0.001
	ISS: II vs I	1.48	(1.04 - 2.11)	0.031		ISS: II vs I	2.09	(1.17 - 3.71)	0.012
	ISS: III vs I	1.86	(1.18 - 2.93)	0.007		ISS: III vs I	3.74	(1.96 - 7.13)	<0.001
	LDH: >ULN vs ≤ULN	1.39	(0.91 - 2.13)	0.129		LDH: >ULN vs ≤ULN	1.4	(0.78 - 2.52)	0.254
	age: ≥60 vs <60	1.07	(0.78 - 1.48)	0.67		age: ≥60 vs <60	0.95	(0.59 - 1.54)	0.834
**1-year maintenance**									
		**HR**	**95% CI**	**p value**			**HR**	**95% CI**	**p value**
	MRD status: neg vs pos	0.32	(0.16 - 0.62)	<0.001		MRD status: neg vs pos	0.37	(0.12 - 1.14)	0.082
	FISH: High vs Standard	3.65	(1.68 - 7.92)	0.001		FISH: High vs Standard	4.51	(1.25 - 16.29)	0.022
	ISS: II vs I	1.29	(0.62 - 2.68)	0.503		ISS: II vs I	2.93	(0.78 - 11.06)	0.112
	ISS: III vs I	2.12	(0.94 - 4.79)	0.070		ISS: III vs I	5.26	(1.21 - 22.87)	0.027
	LDH: >ULN vs ≤ULN	1.19	(0.49 - 2.87)	0.703		LDH: >ULN vs ≤ULN	0.84	(0.19 - 3.76)	0.818
	age: ≥60 vs <60	1.90	(1.06 - 3.41)	0.032		age: ≥60 vs <60	1.98	(0.70 - 5.57)	0.196

Adjusted for sex.

MRD, minimal residual disease; FISH, fluorescence in situ hybridization; ISS, International Staging System; LDH, lactate dehydrogenase; ULN, upper limit of normal; ITT, intention to treat.

Supplementary Figures

Figure 1. A) Progression-free survival in the pre-maintenance population (suspected CR). B) Overall survival in the pre-maintenance population (suspected CR).

A)

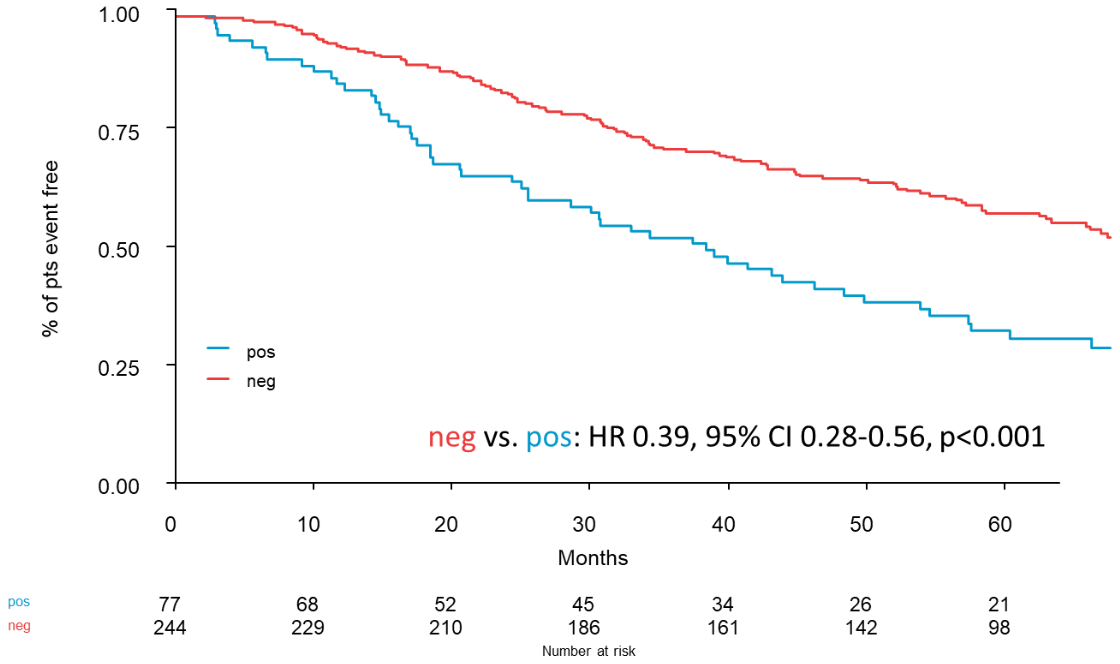

B)

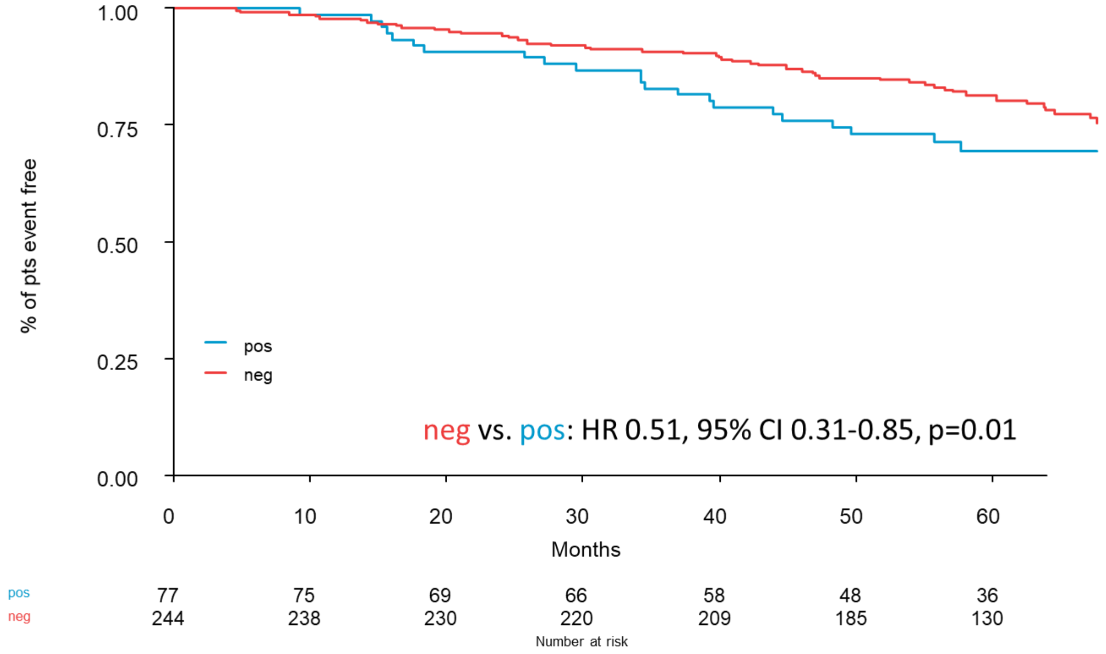

Figure 2. A) Subgroup analysis (pre-maintenance) of progression-free survival. B) Subgroup analysis (pre-maintenance) of overall survival.

A)

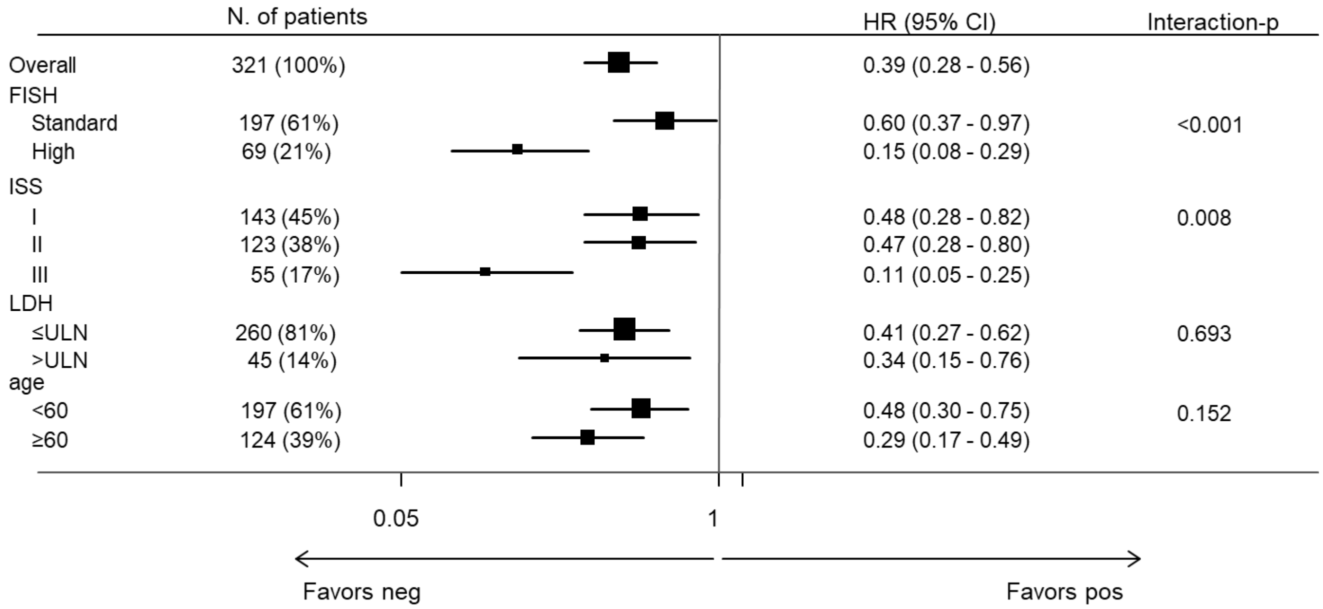

B)

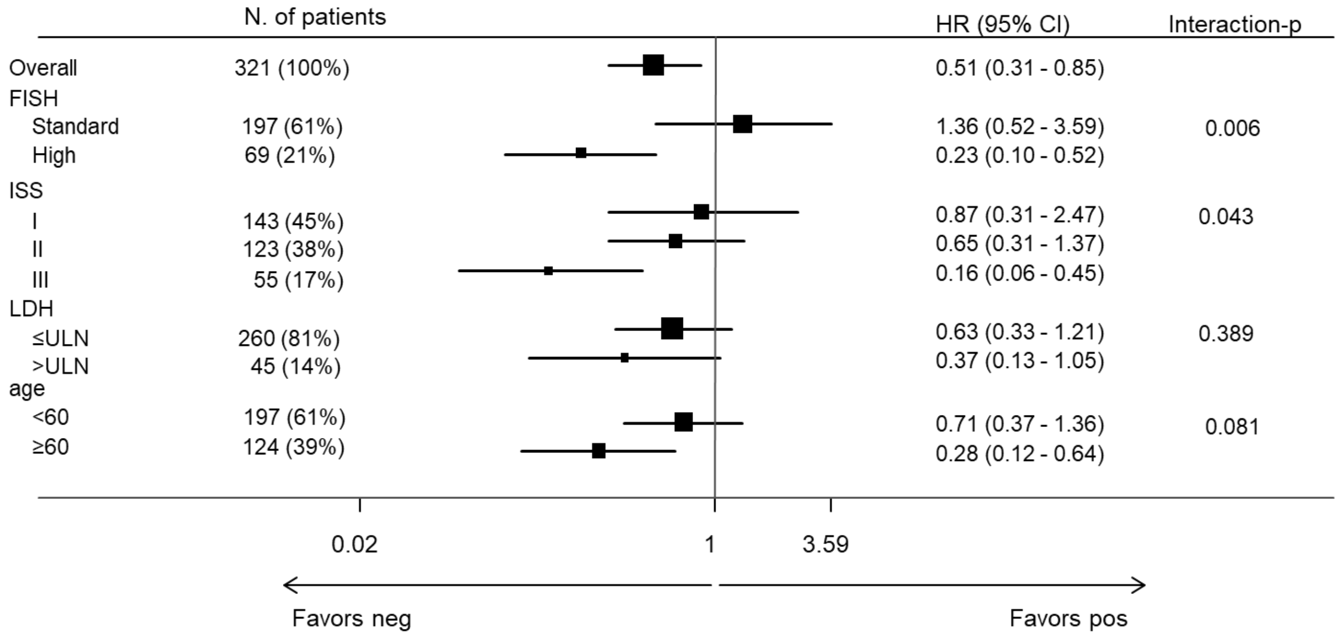

FISH, fluorescence in situ hybridization; ISS, International Staging System; LDH, lactate dehydrogenase; ULN, upper limit of normal.

Figure 3. Outcome in the pre-maintenance MRD population with high-risk features:
A) Progression-free survival in high-risk patients by FISH. B) Overall survival in high-risk patients by FISH. C) Progression-free survival in ISS stage III patients. D) Overall survival in ISS stage III patients.

A)

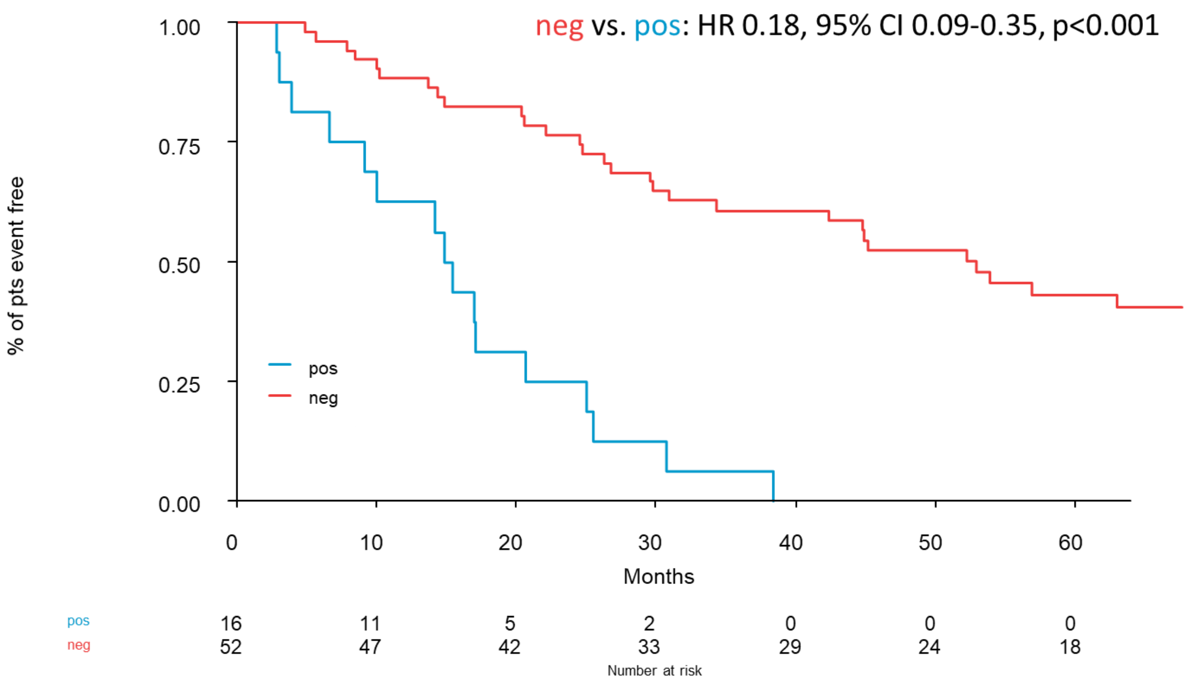

B)

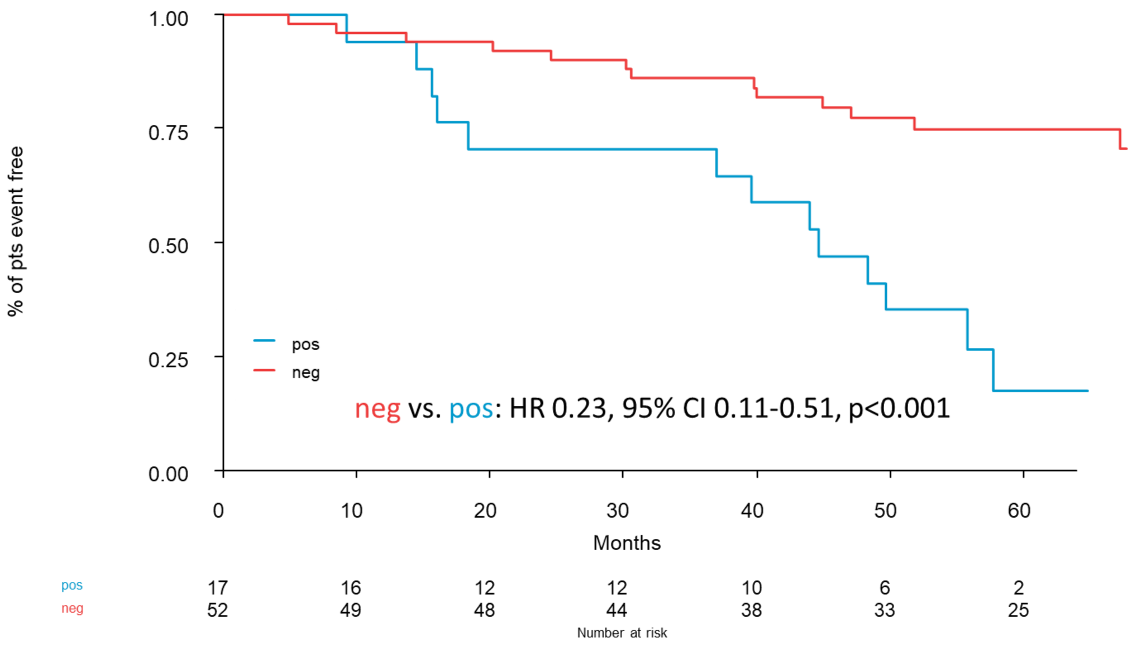

C)

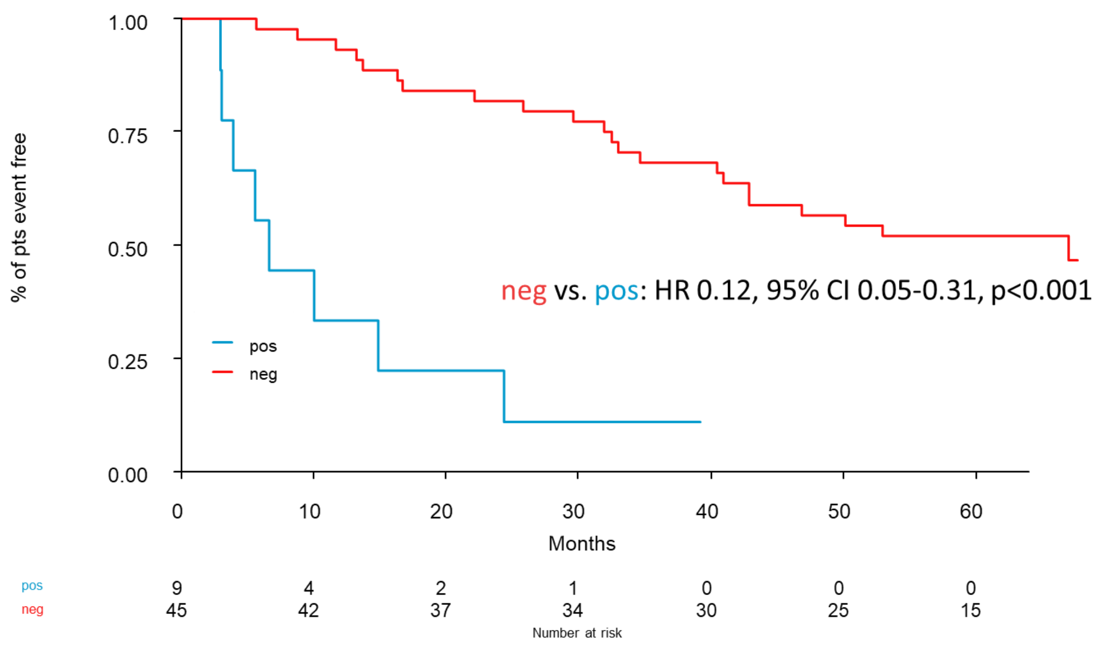

1.00
0.75
0.50
0.25
0.00
% of pts event free
0
10
20
30
40
50
60
Months
neg vs. pos: HR 0.12, 95% CI 0.05-0.31, p<0.001
pos
neg
pos 9 4 2 1 0 0 0
neg 45 42 37 34 30 25 15
Number at risk

D)

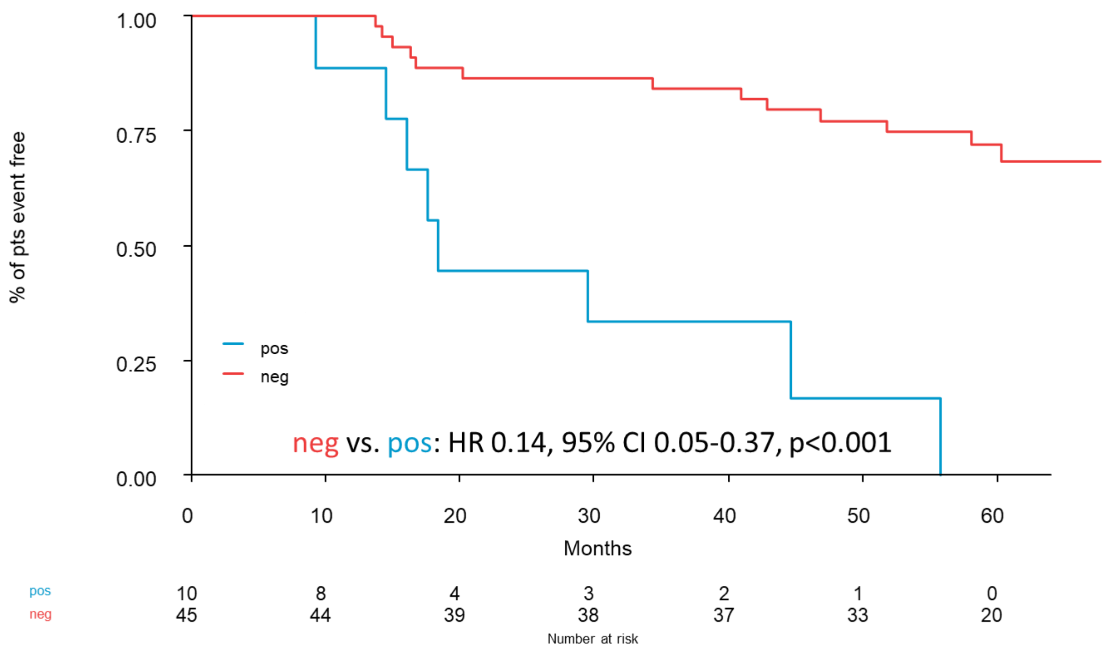

1.00
0.75
0.50
0.25
0.00
% of pts event free
0
10
20
30
40
50
60
Months
neg vs. pos: HR 0.14, 95% CI 0.05-0.37, p<0.001
pos
neg
pos 10 8 4 3 2 1 0
neg 45 44 39 38 37 33 20
Number at risk

Figure 4. Outcome of patients with sustained MRD negativity

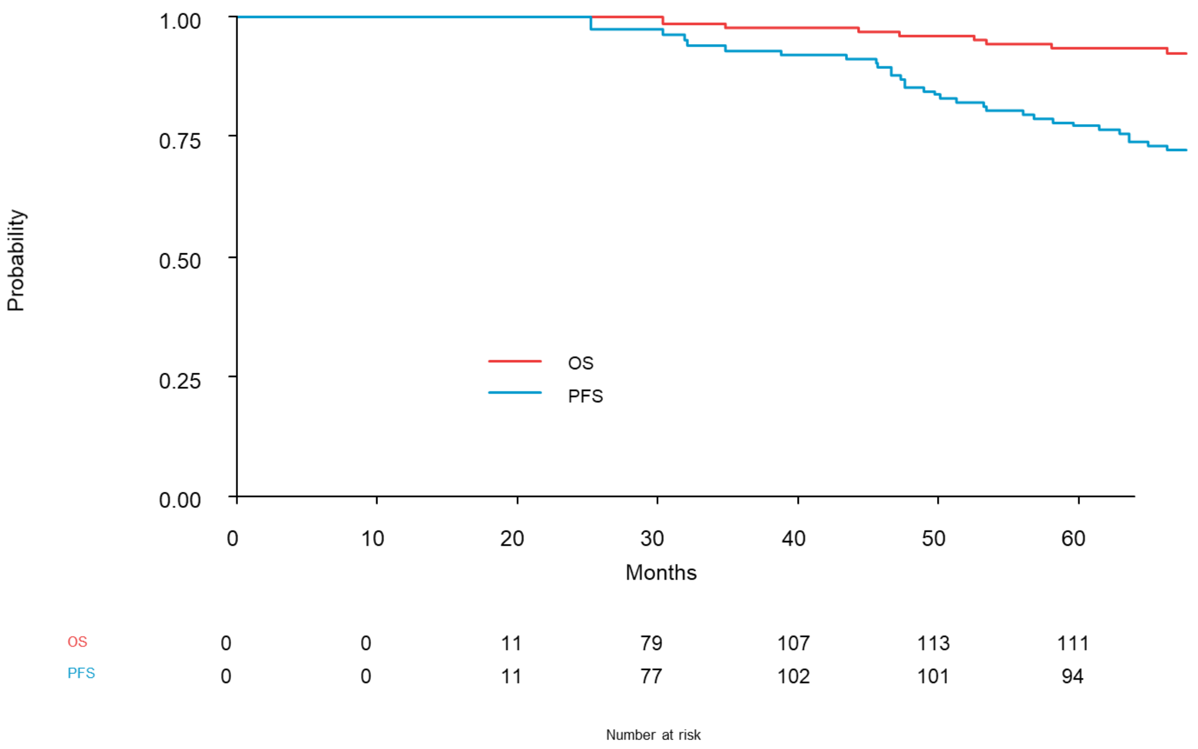

Figure 5. A) Progression-free survival in the 1-year lenalidomide maintenance MRD population. B) Overall survival in the 1-year lenalidomide maintenance MRD population

A)

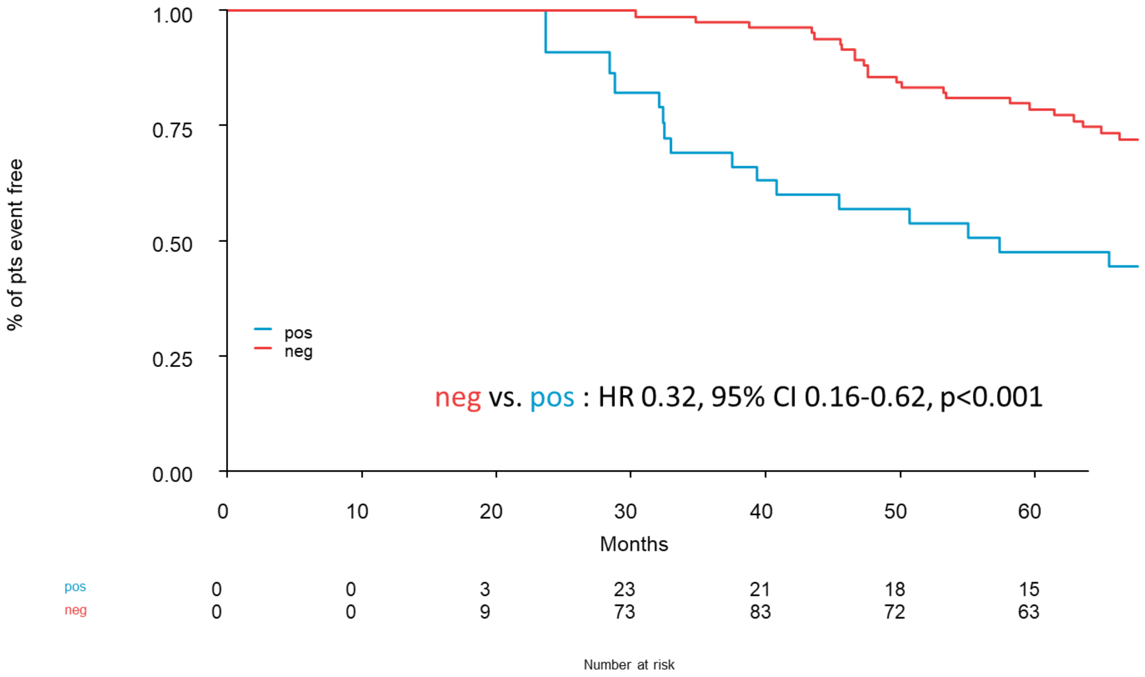

B)

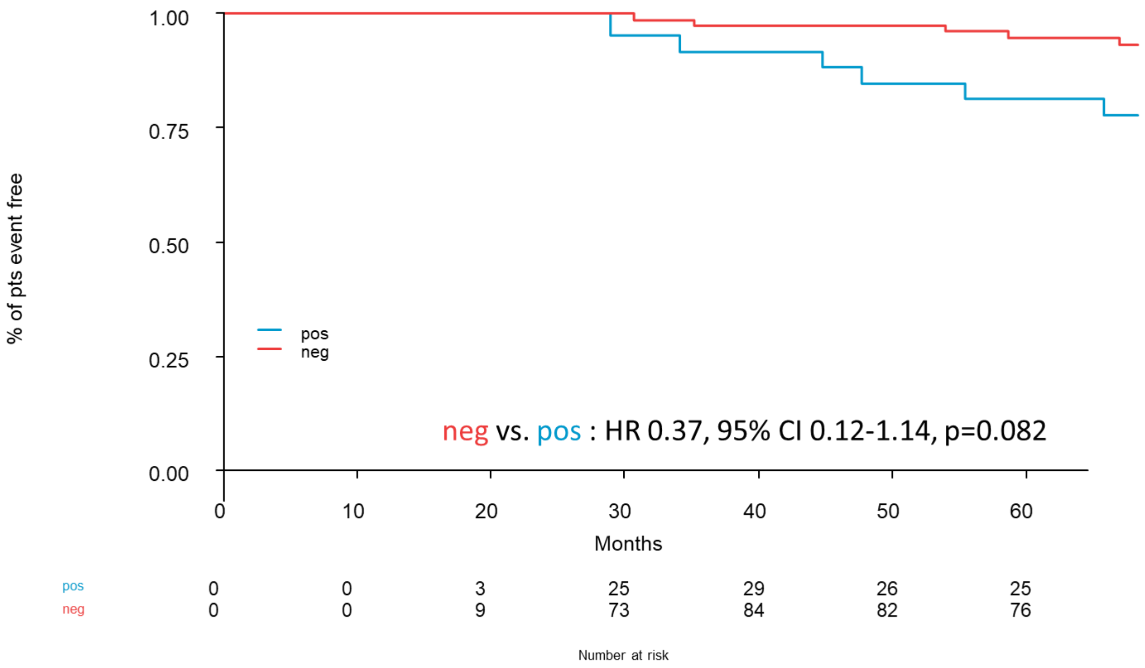

Figure 6. Progression-free survival according to MRD conversion in the 1-year lenalidomide maintenance MRD population.

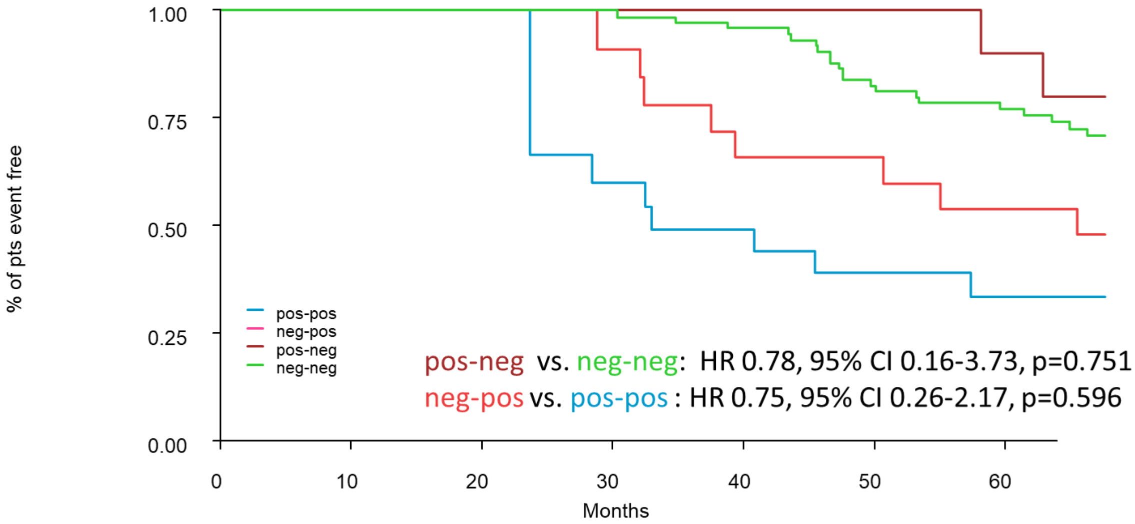

	0	10	20	30	40	50	60
pos-pos	0	0	2	10	10	7	6
neg-pos	0	0	1	13	11	11	9
pos-neg	0	0	1	9	10	10	9
neg-neg	0	0	8	64	73	62	54
